# An Epidemiological Investigation of Male-Female Differences in Drinking and Drinking-Related Problems between US-Born and Foreign-Born Latino and Asian Americans

**DOI:** 10.1155/2013/631912

**Published:** 2012-07-18

**Authors:** Hui G. Cheng, Orla McBride

**Affiliations:** ^1^Shanghai Mental Health Center, Shanghai Jiao Tong University, 3210 Humin Road, Shanghai 201108, China; ^2^Division of Population Health Sciences, Department of Psychology, Royal College of Surgeons in Ireland, 123 Street Stephen's Green, Dublin 2, Ireland

## Abstract

*Background*. It has been widely documented that males were more likely to drinking alcohol and have alcohol use disorders (AUD). The degrees of the male-female differences in drinking and AUD have varied across countries. The reasons behind these variations have not been fully understood. The current study compared the estimated male-female differences across US-born and foreign-born Latino and Asian Americans with respect to alcohol drinking behavior and AUD. *Method*. Data come from the National Latino and Asian American Study (NLAAS), a national household survey of adults with Latinos and Asian decent in the United States. Male-female differences were estimated for drinking behavior and AUD among drinkers for US-born and foreign-born individuals, respectively. Zero-inflated Poisson regressions were utilized to estimate male-female differences in the number of AUD clinical features once it occurs. *Results*. Larger male-female differences were found for foreign-born individuals as compared to US-born individuals, especially the occurrence of AUD among drinkers. Once AUD clinical feature occurs, there was no male-female difference for foreign-born individuals, while there was a males excess in the number of clinical features for US-born individuals. *Conclusion*. Results from this study supports the importance of sociocultural influence in drinking and AUD. Implications for prevention and intervention programs were discussed.

## 1. Introduction

Worldwide, it has been extensively documented that alcohol consumption and alcohol use disorders (AUD) are more common in males as compared to females [1–6]. The reason of this male-female difference is both biological and sociocultural. Biological factors include differences in average volume of body water where ethanol can be distributed, levels of enzymes and their activities to metabolize ethanol, neurotransmitters and receptors through which ethanol affects the brain, and others [7–10]. Sociocultural factors include different levels of stigma attached to female drinking, social expectation toward different gender roles, alcohol availability, and others [11–13]. That is, drinking has been used as a way to express masculinity among males. In contrast, the traditional role for female is family-oriented. Also, female drinking has been associated with higher vulnerability of sexual assault [14, 15]. Thus, female drinking is discouraged socially due to the fear that it may interfere with their responsibilities [15]. These factors interplay with each other leading to the observed male-female differences in drinking and the occurrence of AUD. Despite the consistent finding that males are more likely to experience drinking-related problems, wide variations in male-female differences have been found across cultures and populations and this variation in male-female differences across countries is considered to be too wide to be explained solely by biological differences [4–6, 15, 16]. Besides theoretical value, studies of male-female differences in drinking and AUD are also of public health importance. Some studies have recently documented a possible convergence in male and female drinking patterns in the US, which may in turn increase disease burden associated with alcohol drinking in females (e.g., [17]). Understanding the observed male-female differences may provide useful information for the design of future prevention.

Previous studies have shown that the male-female difference in AUD is considerably smaller in the United States as compared to Latin American and Asian countries, suggesting female drinking is tolerated more in the USA when compared to Latin American and Asian countries [11, 16, 18–22]. For example, a study using data from the National Latino and Asian American Study (NLAAS) found male-female ration in substance use disorders in foreign-born Latinos living in the US was at least 3 times higher compared to their US-born counterparts [6]. Furthermore, among foreign-born individuals drinking problem increases as the number of generations since immigration to the USA as well as the age of immigration decreases [6, 23]. Some research has hypothesized that this difference is due to acculturation process into a society where female drinking is more tolerated. Some studies found supporting evidence that acculturation is positively associated with heavy drinking in females [24, 25], while others found no such association [26, 27]. 

Reviewing previous literature, some exploration may aid further understanding of drinking and AUD. As suggested by previously studies, male-female difference may vary across various stages of alcohol involvement [28, 29]. Thus, comparing male-female difference between US-born and foreign-born individuals with respect to the transition from the earlier alcohol involvement to the occurrence of AUD may provide useful information. Alcohol use disorder includes socially maladaptive drinking (in line with DSM-IV alcohol abuse) and alcohol dependence, both of which involve multiple clinical features. Among AUD cases, the number of clinical features can vary widely. Studies have found that greater numbers of clinical features predict the persistence of AUD [30] and the occurrence of new clinical features [31]. In this context, it is of interest to probe if males are more likely to have more clinical features of AUD as compared to females after the occurrence of AUD. In this context, aims of the current study are (1) to estimate variations in male-female difference in terms of earlier alcohol involvement and the transition from alcohol drinking to the occurrence of AUD comparing US-born to foreign-born Latino and Asian Americans (LAA) (2) to assess if males are more likely to have a greater number of AUD clinical features once it occurs.

## 2. Method

### 2.1. Study Design and Sample Selection

Latinos and Asians are two of the fastest growing ethnic groups within the USA. The US census bureau projects that by 2050, each group will nearly triple in size (e.g., Latinos to 133 million, Asians to 34 million), accounting for more than one-third (37.9%) of the total US population jointly [32]. However, much is unknown about psychiatric disorders among the LAA population. Against this background, the NLAAS was conducted to assess the prevalence and correlates of psychiatric disorders among LAA via standardized interviews and survey methodology [33]. The sampling procedure was designed to yield a nationally representative sample of household-dwelling LAA adults, aged 18 years and older. The sampling approach consisted of multistage nationwide probability sampling and supplemental sampling from high density geographical areas of LAA. High-density supplemental samples were added to the nationwide sampling to optimize the statistical efficiency. One eligible adult was randomly selected as main respondent from each identified household. In a subsample, one additional adult was selected as second respondent. The sampling procedure resulted in 4345 main respondents and 1234 second respondents. Of all 5579 identified individuals, 4649 individuals completed interview. Weighted response levels were 75.7% and 80.3% among main respondents and second respondents, respectively. Comprehensive details about the samples and calculation of response levels are available elsewhere [33]. The study protocol was reviewed and approved by the cognizant institutional review board for protection of human subjects in research. Data collection took place during May 2002 to December 2003.

## 3. Measures

The study utilized a version of the World Health Organization's (WHO) World Mental Health Composite International Diagnostic Interview (WMH-CIDI) [34]. The WMH-CIDI is a comprehensive fully structured diagnostic interview designed to be administered by trained lay interviewers for the assessment of mental disorders according to DSM-IV criteria. To minimize the number of interview refusals due to insufficiency with the English language, the CIDI was translated into three other languages: Spanish, Chinese, and Vietnamese. Respondents were interviewed in their own homes. 

In this study, key response variables are lifetime history of AUD including DSM-IV alcohol abuse and alcohol dependence. The endorsement of any of the four clinical features of socially maladaptive drinking qualifies the individual of lifetime history of DSM-IV alcohol abuse. To ease discussion and presentation, they were labeled as “responsibility interference,” “drink despite social problems”, “hazardous use”, and “legal problems.” Lifetime DSM-IV alcohol dependence was defined as the occurrence of at least three of the seven clinical features of alcohol dependence during the same 12 months. Lifetime occurrence of clinical features of alcohol dependence is defined as the occurrence of at least one of the seven DSM-IV clinical features and “irresistible desire to drink.” Opportunity to drink alcohol is defined as ever having opportunity to drink alcohol; more than minimum (MTM) drinking is defined as having at least 12 drinks in a given year; heavier drinking is defined as at least 5 drinks per day for males and 4 for females. This latter definition is consistent with the definition of “binge drinking” used by the US National Institute of Alcohol Abuse and Alcoholism (NIAAA) and the most commonly used definitions for “binge drinking” and “heavy episodic drinking” in previous studies [35].

Gender and place of birth were the main covariates in this analysis. Other covariates include age (in years), ethnicity (Latino or Asian), and number of parents born in the USA. All of these variables were based upon the respondent's self-report. 

## 4. Analysis

During the first steps, we provided a description of the sample and lifetime estimates of AUD, as well as stratified estimates by male-female and place of birth. In subsequent steps, a series of logistic regressions were conducted to estimate the variation in male-female differences in alcohol involvement and the occurrence of AUD among drinkers. The male-female gap in drinking and AUD is estimated in the form of odds ratios (ORs). Statistical comparisons are made to investigate the variation of OR in US-born and foreign-born LAA by the inclusion of a product term. First, bivariate logistic regression was conducted to produce the unadjusted OR. Second, age, ethnicity, and number of parents born in the US, was introduced to the regression model. (The variable “number of grandparents born in the US” was initially included as well, but it is not associated with the outcomes. It was not included in the final models in order to obtain a parsimonious model.) In these steps, since the occurrence of outcomes, especially early alcohol involvement (e.g., opportunities to drink and trying alcohol when given the opportunity) was quite high, the odds ratio (OR) is not a good simulation for relative risk (RR). In order to be able to attach a more substantive meanings to estimates, we used the formula suggested by Zhang and Yu [36] to transform the OR into RR. 

The final steps consisted of using the zero-inflated Poisson (ZIP) regressions to estimate male-female differences for foreign-born and US-born LAA with respect to both the occurrence of AUD and the increment in numbers of clinical features of AUD among drinkers. Due to the fact that a vast majority of individuals did not have any clinical feature of AUD (a count of zero) there was an “over-dispersion” of zeros in the outcome, the number of AUD clinical features. The ZIP model can account for this “inflation” of zeros [37]. The ZIP model assumes that there are two groups of individuals, one consists of those who never have a value greater than zero (the zero group), another consists of those who have a value greater than 0 (the nonzero group). This assumption is appropriate in this context because the majority of the LAA population never had any clinical feature of AUD due to characteristics such as light or moderate drinking. The ZIP model produces two sets of estimates: one set of estimates to associate the likelihood of being in the nonzero group (or the zero group), the other set to associate covariates with the increment of outcome given the nonzero group. The main covariate was gender. Other covariates included age, ethnicity, and number of parents born in the USA for adjustment.

The complex survey sampling strategy and selection probabilities were accounted for by applying proper sampling weights and post stratification adjustments. In this work, the precision of estimates was expressed by 95% confidence intervals. Stata software was used for all analyses (version 9.2, Stata Corporation, 2009). 

## 5. Results

### 5.1. The Sample Description

The sample included 1378 US-born LAA (924 Latinos and 454 Asians) and 3268 foreign-born LAA (1629 Latinos and 1659 Asians). With respect to alcohol use and related problems, males were more likely to drink and were more likely to have drinking-related problems than females in both US-born and foreign-born LAA (see Table 1). 

US-born Individuals were more likely to drink when compared to foreign-born individuals (86.8% and 63.4% for US-born males and females, resp., 75.7% and 30.3% for foreign-born males and females, resp.). The same pattern was found for the lifetime occurrence of AUD. 

Smaller male-female ratios were found for US-born LAA in the occurrence of AUD as compared to foreign-born LAA: the male-female ratio was above 20 (e.g., 8.3% and 0.4% for socially maladaptive drinking for males and females, resp.) for foreign-born LAA and approximately three in US-born LAA. 

Table 2 presents estimated relative risks ratios (RRs) in relation to the transition from opportunity toward the occurrence of AUD for foreign-born and US-born LAA, respectively. For each outcome studied here, statistically significant variation in RR was found across foreign-born LAA and US-born LAA (*P* < 0.05). Larger RR were found for the foreign-born LAA. Nonetheless, point estimates indicate that the male excessive risk is rather small in earlier alcohol involvement (i.e., the opportunity to drink alcohol and tried alcohol provided opportunities) in both groups. In contrast, as alcohol drinking progresses, much larger male-female gaps were found for foreign-born LAA (e.g., from 1.3 for alcohol opportunity to 8.7 for DSM-IV alcohol abuse among MTM drinkers), while moderately larger male-female gaps were found for US-born LAA (e.g., from 1.1 for alcohol opportunity to 2.0 for DSM-IV alcohol abuse among MTM drinkers). In foreign-born LAA, as one female drinker developed any socially maladaptive drinking, there were almost 10 male drinkers did so; in US-born LAA, this ratio was two. Similar pattern was found in the occurrence of alcohol dependence. Interestingly, the male excessive risk of heavy drinking, which is an indicator of problematic drinking, was much smaller, especially in foreign-born LAA. Variations across Latinos and Asians were tested, but the results were not statistically significant (*P* > 0.05).

As presented in Table 3, the male-female difference for foreign-born LAA lies mainly in the occurrence of clinical feature of AUD (adjusted OR = 9.5, 95% CI = 4.3, 21.0); once AUD clinical feature occurs, there was no male-female difference anymore with respect to the increment in the number of clinical features. In contrast, for US-born LAA, males were more likely to have any AUD clinical feature as compared to females (adjusted OR = 3.6, 95% CI = 2.5, 5.1). Among individuals who had ever experienced at least one clinical feature, males were more likely to experience a greater number of AUD clinical features overall.

## 6. Discussion

The main findings of this study maybe summarized succinctly. First, substantially larger male-female differences in drinking and AUD were found for foreign-born LAA compared to their US-born counterparts. Second, the male-female difference in foreign-born LAA occurs mainly in later stages that involve the occurrence of AUD. In contrast, the male-female difference is much more homogeneous across all stages of alcohol involvement among US-born LAA. Third, in foreign-born LAA, once AUD clinical feature occurs, there was no male-female in the increment in number of clinical features; for US-born LAA, males were more likely to have a larger number of AUD clinical features once it occurs.

The current study found male-female ratios in foreign-born LAA were close to those from Latin and Asian countries [16, 18–21], while the male-female ratio in US-born LAA was almost the same as estimates for the US population [38]. These estimates were not due to differential distribution of age, ethnicity or “the number of parents born in the US.” Given that the male-female differences were much similar at the early stages of alcohol involvement as compared to later stages (i.e., when maladaptive drinking occurred) across foreign- and US-born LAA, it is unlikely that the observed male-female differences in alcohol involvement is solely due to biological variations. Findings from this study correspond with previous knowledge and provide further evidence about male-female differences in drinking behavior highlighting the importance of social-environmental factors in alcohol drinking behavior and the occurrence of AUD [6, 11, 15, 16]. Indeed, the finding that males were more likely to have opportunity to drink alcohol is a direct piece of evidence about the social-environmental impact on alcohol involvement. Based on the United Nation Development Programme (UNDP)'s report, United State outranked most of the Latin American and Asian countries, especially those contributed large numbers of immigrants to the USA, in gender-related human development Indices, such as gender-related development index (GDI), and gender empowerment measure (GEM) [39]. The former is a gender inequality adjusted index to measure social and economic development of a country. The latter evaluates women's participation in politics and economics. As such, the convergence in social role expectation between males and females would have resulted in a convergence in alcohol drinking, which is traditionally viewed as a demonstration of masculine, including over-drinking. Another possibly important underlying mechanism is acculturation which may influence female drinking behavior more compared to males [24, 25]. 

This study provided some empirical evidence on the male-female differences for US-born and foreign-born LAAs in relation to stages of alcohol involvement and what happen after the occurrence of the first clinical feature of AUD. The results signal that the differential social expectation may impact drinking behavior more in the later stages when maladaptive drinking pattern is manifested rather than early stages of alcohol involvement. It is interesting that the male-female difference in heavy drinking is not as large as that for AUD after MTM drinking has occurred. This suggests that occasional heavy episodic drinking in female may not be socially discouraged behavior as long as it does not evolve into a pattern of maladaptive drinking. However, heavy episodic drinking is associated with substantial disease burden through various routes [40]. The results call for attention to the importance of intervention of female heavy drinking. The distinct pattern in the increment of clinical features of AUD after the fist clinical feature has occurred across foreign-born and US-born LAA is novel. It is possible that for foreign-born LAA, female drinkers have to “cross a higher hurdle” to manifest a maladaptive drinking pattern (compared to US-born LAA) due to strong traditional social expectations toward females. Once the hurdle is crossed the impact of social expectation is not relevant to female drinkers anymore, and this results in the observed absence of male-female difference in the increment of AUD clinical features.

Several study limitations merit attention while interpreting findings. Of central concern is the cross-sectional nature of the study. Possible recall bias and survival bias cannot be ruled out [41]. Thus, the male-female difference in the transition of different alcohol involvement stages is best understood as the reported male-female difference in alcohol involvement stages among survivors. Nonetheless, there has been no evidence for a male-female associated differential reporting of drinking. All estimates were adjusted for age as well. With respect to the population under study, institutional people and adolescents were not included in this study. Thus, the results cannot be generalized to these populations. With respect to sample size, although this study has the largest sample size of LAA, there was limited number of AUD cases in the foreign-born female group (*n* = 11 as presented in Table 1). Thus, the estimates for the foreign-born LAA may not be precise. This also precluded the possibility to study acculturation variables (e.g., language efficiency) or the cross-ethnic variations in this study. Nonetheless, the robust variations in male-female ratios between foreign-born LAA and US-born LAA were not likely to be due to chance. With respect to reporting bias, it is possible that foreign-born females held higher level of self-stigma toward drinking and AUD as compared to US-born females [11, 12]. Unfortunately, to our knowledge, there has been no study about the degree and direction that self-stigmatization might influence reporting in a survey context. Regarding the assessment, although CIDI interviews were conducted using four languages, it is possible that some interviewees did not understand questions fully due to insufficient language ability given the multilinguistic nature of the LAA population. Last but not least, information about drinking levels before the occurrence of AUD cannot be obtained, although it holds great relevance in understanding the natural history of drinking problems. Future prospective studies with little attrition are needed to address this issue. 

Counterbalancing strengths include that NLAAS is a nationally representative study. With statistical adjustments, such as sample weights and poststratification adjustment, the selection bias was minimized and thus results were generalizable to the LAA population as a whole. Based on findings like these, it is of interest to probe the role of acculturation in order to shed some light on the mechanism of the observed male-female differences among foreign-born LAAs. Also, studies on the male-female differences stratified by fine-grained grouping of ethnicity (e.g., different Asian and Latino ethnicities) may yield more relevant results for each subgroups of LAA.

## Figures and Tables

**Table 1:** Description of sociodemographic characteristics and drinking-related outcomes in males and females. Data from NLAAS, 2002-2003.

		The entire sample			US-born				Foreign-born			
Demographics					Males		Females		Males		Females	
		*n* of cases	Mean^1^	se	*n* of cases	Mean^1^	*n* of cases	Mean^1^	*n* of cases	Mean^1^	*n* of cases	Mean^1^
Age (years)		4649	38.9	0.5	629	35.9	749	37.8	1494	39.0	1774	41.2

		*n*	%^1^	se	*n*	%^1^	*n*	%^1^	*n*	%^1^	*n*	%^1^

Race/Ancestry	Hispanic	2554	73.6	2.0	403	83.6	521	83.1	723	70.1	906	65.71
	Asian	2095	26.5		226	16.4	228	16.9	771	29.9	868	34.29
Born in the US	Yes	1378	36.6	2.0								
	No	3268	63.4									
Alcohol drinking and alcohol use disorders												
MTM drinking^2^		3730	61.2	1.2	540	86.8	484	63.4	1062	75.7	528	30.3
Past year drinking		1332	32.4	1.2	326	56.0	220	27.2	587	43.0	197	10.8
Ever DSM-IV alcohol abuse		299	8.7	0.9	130	23.8	61	8.7	97	8.2	11	0.4
Ever DSM-IV alcohol dependence		107	3.4	0.4	46	8.8	26	3.3	30	3.5	5	0.1
Ever dependence clinical feature		267	8.0	0.9	119	22.0	53	7.8	85	7.4	10	0.3

^1^Weighted percentage.

^2^MTM: more than minimum drinking.

**Table 2:** Male-female relative risks for transitions of alcohol drinking and AUD stratified by the place of birth. Data from NLAAS, 2002-2003.

	Foreign-born								US-born						
		wt%^1^	uRR	95% C.I.		aRR^2^	95% C.I.		wt%^1^	uRR	95% C.I.		aRR^2^	95% C.I.	
Opportunities	Female	76.7							94.0						
	Male	95.9	1.3	1.2	1.3	1.2	1.2	1.3	98.9	1.1	1.0	1.1	1.1	1.0	1.1
Tried alcohol given opportunity (*n* = 4098)	Female	84.5							92.3						
	Male	95.0	1.1	1.1	1.1	1.1	1.1	1.1	95.1	1.0	1.0	1.1	1.0	1.0	1.1
MTM drinking among those who tried (*n* = 3708)	Female	47.5							73.9						
	Male	83.4	1.8	1.7	1.8	1.8	1.7	1.8	92.4	1.3	1.2	1.3	1.3	1.2	1.3
Heavy drinking among MTM drinkers (*n* = 2617)	Female	18.2							39.1						
	Male	41.4	2.3	1.9	2.7	2.3	1.9	2.7	53.3	1.4	1.2	1.5	1.4	1.2	1.6
DSM-IV alcohol abuse among MTM drinkers	Female	1.3							14.3						
	Male	11.0	8.7	4.1	16.9	8.7	4.2	16.8	28.5	2.0	1.6	2.5	2.1	1.7	2.6
DSM-IV alcohol dependence among MTM drinkers	Female	0.4							5.2						
	Male	4.6	12.4	4.5	32.7	12.2	4.4	32.4	10.1	1.9	1.2	3.0	2.0	1.3	3.1
Any alcohol dependence clinical feature among MTM drinkers	Female	1.1							12.4						
	Male	9.8	8.8	4.0	18.2	8.8	4.1	18.0	25.3	2.1	1.6	2.6	2.2	1.7	2.7

^1^Weighted percentage.

^2^Estimates were adjusted for age, number of parents born in USA, and ethnicity.

**Table 3:** Estimates for male-female ratios from the zero-inflated poisson regression among drinkers. Data from NLAAS, 2002-2003.

	Foreign-born LAA		US-born LAA	
	Model 1^1^	Model 2^1^	Model 1^1^	Model 2^1^
OR to be in the nonzero group (95% CI)	9.4 (4.2, 21.0)	9.5 (4.3, 21.0)	2.4 (1.7, 3.4)	2.6 (1.9, 3.6)
*P* value	<0.001	<0.001	<0.001	<0.001
Coefficient for the increment in the count of clinical features (95% CI)	0.17 (−0.13, 0.46)	0.21 (−0.12, 0.53)	0.35 (0.02, 0.68)	0.39 (0.03, 0.70)
*P* value	0.26	0.22	0.04	0.03

^1^Model 1 adjusted for age and ethnicity; model 2 additionally adjusted for number of parents born in the USA.

## References

[1] Rahav G, Wilsnack R, Bloomfield K, Gmel G, Kuntsche S (2006). The influence of societal level factors on men’s and women’s alcohol comsumption and alcohol problems. *Alcohol and Alcoholism*.

[2] Lopez AD, Mathers CD, Ezzati M, Jamison DT, Murray CJ (2006). Global and regional burden of disease and risk factors, 2001: systematic analysis of population health data. *The Lancet*.

[3] Zilberman M, Tavares H, El-Guebaly N (2003). Gender similarities and differences: the prevalence and course of alcohol- and other substance-related disorders. *Journal of Addictive Diseases*.

[4] Vega WA, Canino G, Cao Z, Alegria M (2009). Prevalence and correlates of dual diagnoses in U.S. Latinos. *Drug and Alcohol Dependence*.

[5] Alegría M, Canino G, Shrout PE (2008). Prevalence of mental illness in immigrant and non-immigrant U.S. Latino groups. *American Journal of Psychiatry*.

[6] Canino G, Vega WA, Sribney WM, Warner LA, Alegría M (2008). Social relationships, social assimilation, and substance use disorders among adult Latinos in the U.S.. *Journal of Drug Issues*.

[7] Devaud LL, Risinger FO, Selvage D (2006). Impact of the hormonal milieu on the neurobiology of alcohol dependence and withdrawal. *Journal of General Psychology*.

[8] Ely M, Hardy R, Longford NT, Wadsworth MEJ (1999). Gender differences in the relationship between alcohol consumption and drink problems are largely accounted for by body water. *Alcohol and Alcoholism*.

[9] Yoshida A (1994). Genetic polymorphisms of alcohol metabolizing enzymes related to alcohol sensitivity and alcoholic diseases. *Alcohol and Alcoholism*.

[10] Goedde HW, Agarwal DP (1987). Polymorphism of aldehyde dehydrogenase and alcohol sensitivity. *Enzyme*.

[11] Bloomfield K, Gmel G, Neve R, Mustonen H (2001). Investigating gender convergence in alcohol consumption in Finland, Germany, The Netherlands, and Switzerland: a repeated survey analysis. *Substance Abuse*.

[12] Holmila M, Raitasalo K (2005). Gender differences in drinking: why do they still exist?. *Addiction*.

[13] Isidore S, Obot RR (2005). *Alcohol, Gender, and Drinking Problems. Perspectives from Low and Middle Income Countries*.

[14] Christie-Mizell CA, Peralta RL (2009). The gender gap in alcohol consumption during late adolescence and young adulthood: gendered attitudes and adult roles. *Journal of Health and Social Behavior*.

[15] Wilsnack RW, Wilsnack SC, Kristjanson AF, Vogeltanz-Holm ND, Gmel G (2009). Gender and alcohol consumption: patterns from the multinational GENACIS project. *Addiction*.

[16] Wilsnack RW, Vogeltanz ND, Wilsnack SC (2000). Gender differences in alcohol consumption and adverse drinking consequences: cross-cultural patterns. *Addiction*.

[17] Keyes KM, Grant BF, Hasin DS (2008). Evidence for a closing gender gap in alcohol use, abuse, and dependence in the United States population. *Drug and Alcohol Dependence*.

[18] Obot IS, Room R (2005). *Alcohol, Gender and Drinking Problems*.

[19] Kawakami N, Shimizu H, Haratani T, Iwata N, Kitamura T (2004). Lifetime and 6-month prevalence of DSM-III-R psychiatric disorders in an urban community in Japan. *Psychiatry Research*.

[20] Medina-Mora ME, Borges G, Benjet C, Lara C, Berglund P (2007). Psychiatric disorders in Mexico: lifetime prevalence in a nationally representative sample. *British Journal of Psychiatry*.

[21] Park JT, Kim BG, Jhun HJ (2008). Alcohol consumption and the CAGE questionnaire in Korean adults: results from the second Korea National Health And Nutrition Examination Survey. *Journal of Korean Medical Science*.

[22] Bloomfield K (2005). *Gender, Culture and Alcohol Problems: A Multi-National Study. Project Final Report*.

[23] Peña JB, Wyman PA, Brown CH (2008). Immigration generation status and its association with suicide attempts, substance use, and depressive symptoms among Latino adolescents in the USA. *Prevention Science*.

[24] Caetano R, Ramisetty-Mikler S, Wallisch LS, McGrath C, Spence RT (2008). Acculturation, drinking, and alcohol abuse and dependence among Hispanics in the Texas-Mexico border. *Alcoholism*.

[25] Vaeth PA, Caetano R, Rodriguez LA (2012). The Hispanic Americans Baseline Alcohol Survey (HABLAS): the association between acculturation, birthplace and alcohol consumption across Hispanic national groups. *Addictive Behaviors*.

[26] Caetano R, Vaeth PAC, Rodriguez LA (2012). The Hispanic Americans baseline alcohol survey (HABLAS): acculturation, birthplace and alcohol-related social problems across Hispanic national groups. *Hispanic Journal of Behavioral Sciences*.

[27] Ehlers CL, Gilder DA, Criado JR, Caetano R (2009). Acculturation stress, anxiety disorders, and alcohol dependence in a select population of young adult Mexican Americans. *Journal of Addiction Medicine*.

[28] Kalaydjian A, Swendsen J, Chiu WT (2009). Sociodemographic predictors of transitions across stages of alcohol use, disorders, and remission in the National Comorbidity Survey Replication. *Comprehensive Psychiatry*.

[29] Lee S, Guo WJ, Tsang A (2009). Associations of cohort and socio-demographic correlates with transitions from alcohol use to disorders and remission in metropolitan China. *Addiction*.

[30] Culverhouse R, Bucholz KK, Crowe RR (2005). Long-term stability of alcohol and other substance dependence diagnoses and habitual smoking: an evaluation after 5 years. *Archives of General Psychiatry*.

[31] Schuckit MA, Smith TL, Landi NA (2000). The 5-year clinical course of high-functioning men with DSM-IV alcohol abuse or dependence. *American Journal of Psychiatry*.

[32] United States Census Bureau (2008). *U.S. Population Projections*.

[33] Heeringa SG, Wagner J, Torres M, Duan N, Adams T, Berglund P (2004). Sample designs and sampling methods for the Collaborative Psychiatric Epidemiololgy Studies (CPES). *International Journal of Methods in Psychiatric Research*.

[34] Kessler RC, Ustün TB (2004). The world mental health (WMH) survey initiative version of the world health organization (WHO) composite international diagnostic interview (CIDI). *International Journal of Methods in Psychiatric Research*.

[35] NIAAA (2004). NIAAA council approves definition of binge drinking. *NIAAA Newsletter*.

[36] Zhang J, Yu KF (1998). What’s the relative risk? A method of correcting the odds ratio in cohort studies of common outcomes. *Journal of the American Medical Association*.

[37] Lambert D (1992). Zero-inflated poisson regression, with an application to defects in manufacturing. *Technometrics*.

[38] Grant BF, Dawson DA, Stinson FS, Chou SP, Dufour MC, Pickering RP (2004). The 12-month prevalence and trends in DSM-IV alcohol abuse and dependence: United States, 1991-1992 and 2001-2002. *Drug and Alcohol Dependence*.

[39] United Nations Development Programme (2007). *The Human Development Report*.

[40] Organization WH (2004). *WHO Global Status Report on Alcohol 2004*.

[41] Caldwell TM, Rodgers B, Power C, Clark C, Stansfeld SA (2006). Drinking histories of self-identified lifetime abstainers and occasional drinkers: findings from the 1958 British Birth Cohort Study. *Alcohol and Alcoholism*.

